# Met receptor is essential for MAVS-mediated antiviral innate immunity in epithelial cells independent of its kinase activity

**DOI:** 10.1073/pnas.2307318120

**Published:** 2023-09-25

**Authors:** Ryu Imamura, Hiroki Sato, Dominic Chih-Cheng Voon, Takayoshi Shirasaki, Masao Honda, Makoto Kurachi, Katsuya Sakai, Kunio Matsumoto

**Affiliations:** ^a^Division of Tumor Dynamics and Regulation, Cancer Research Institute, Kanazawa University, Kanazawa 920-1192, Japan; ^b^The World Premier International Research Center Initiative (WPI)-Nano Life Science Institute, Kanazawa University, Kanazawa 920-1192, Japan; ^c^Innovative Cancer Model Research Unit, Institute for Frontier Science Initiative, Kanazawa University, Kanazawa 920-1192, Japan; ^d^Department of Clinical Laboratory Medicine, Kanazawa University, Graduate School of Medical Science, Kanazawa 920-8641, Japan; ^e^Department of Gastroenterology, Kanazawa University, Graduate School of Medical Science, Kanazawa 920-8641, Japan; ^f^Department of Molecular Genetics, Kanazawa University, Graduate School of Medical Science, Kanazawa 920-8640, Japan

**Keywords:** met receptor, MAVS, RIG-I, inflammatory cytokine, RNA virus

## Abstract

Epithelial tissue is at the forefront of innate immunity, playing a crucial role in the recognition and elimination of pathogens. Met is a receptor tyrosine kinase that is necessary for epithelial cell survival, proliferation, and regeneration. Here, we showed that Met is essential for the induction of cytokine production by cytosolic nonself double-stranded RNA through retinoic acid–inducible gene-I-like receptors (RLRs) in epithelial cells. Surprisingly, the tyrosine kinase activity of Met was dispensable for promoting cytokine production. Rather, the intracellular carboxy terminus of Met interacted with mitochondrial antiviral-signaling protein (MAVS) in RLR-mediated signaling to directly promote MAVS signalosome formation. These studies revealed a kinase activity–independent function of Met in the promotion of antiviral innate immune responses, defining dual roles of Met in both regeneration and immune responses in the epithelium.

Emerging viral infections continue to pose a major threat to global public health. The intracellular viral RNAs are detected by host cellular pattern recognition receptors, a retinoic acid–inducible gene I (RIG-I), melanoma differentiation–associated gene 5 (MDA5) (1), and TLR3 (2). Upon sensing viral RNA, RIG-I and MDA5 undergo oligomerization to recruit the signaling adapter mitochondrial antiviral-signaling protein (MAVS, also known as IFN-β promoter stimulator 1 (IPS-1), Virus-Induced Signaling Adaptor (VISA), CARD adaptor inducing IFN-β (CARDIF)) on the mitochondrial outer membrane (3–5). MAVS develops an oligomerized MAVS signalosome at mitochondria that activates TANK-binding kinase 1 (TBK1) and I kappa B kinase epsilon, drives nuclear translocation of nuclear factor-κB and interferon regulatory factor 3 (IRF3)/IRF7, and eventually leads to the production of type I interferons and proinflammatory cytokines (3–5).

The Met receptor is a transmembrane tyrosine kinase receptor expressed predominantly in epithelial cells and neurons and plays a pivotal role in tissue regeneration after injury (6). Upon hepatocyte growth factor (HGF) binding, the Met receptor stimulates the proliferation, scattering, morphogenesis, and survival of epithelial cells and neurons (6, 7). However, the molecular mechanism by which the epithelium regulates immune response and the involvement of HGF-Met remains undetermined.

## Results and Discussion

**Fig. 1.**
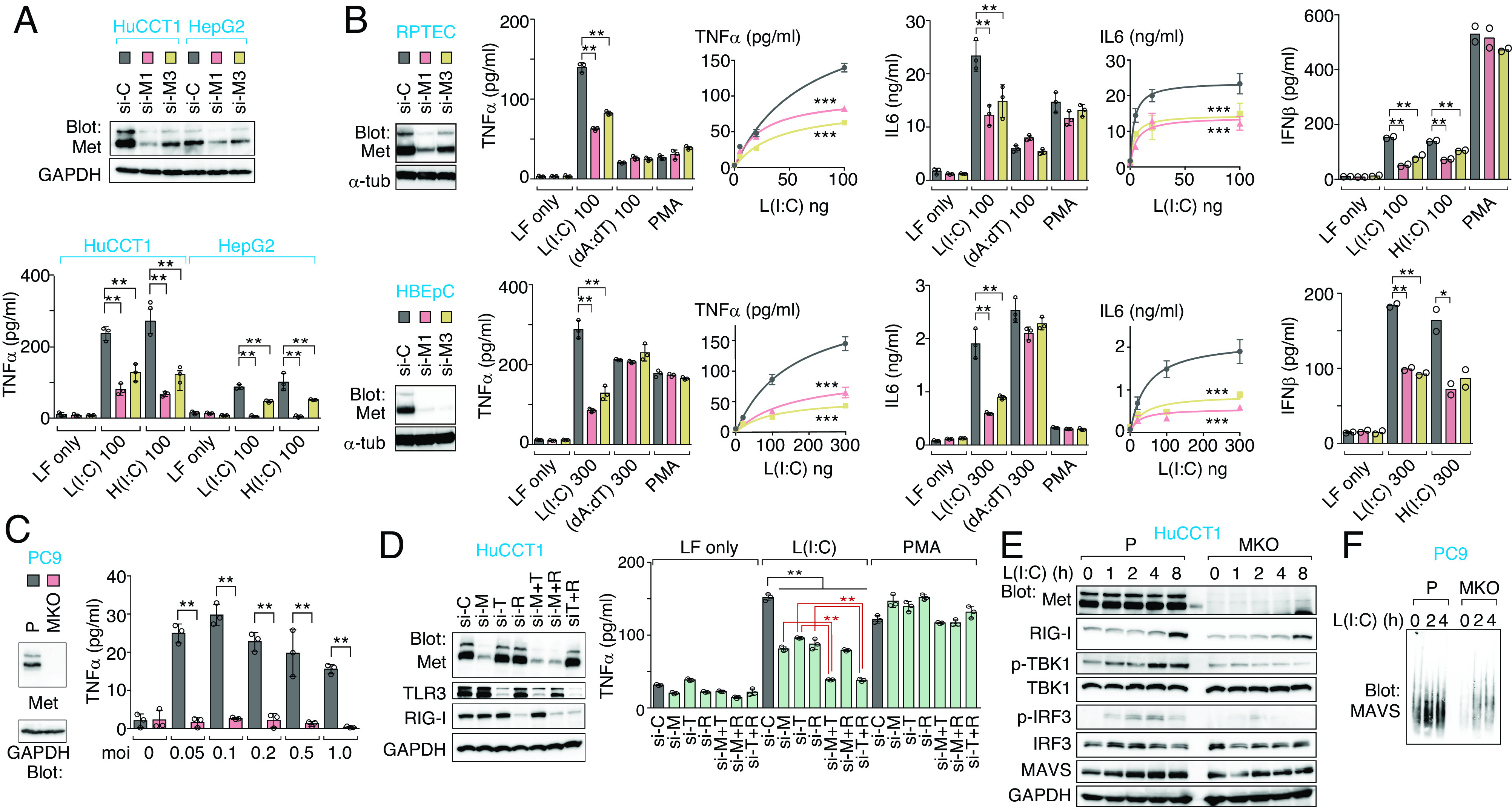
Met is essential for cytokine production through RIG-I-MAVS pathway. (*A* and *B*) Met expression in Met knockdown (si-M) or scrambled siRNA (si-C) in HuCCT1 cells, HepG2 cells (*A*), RPTEC, and HBEpC (*B*) was determined by western blotting. Cells were treated with transfection reagent (LF only), LMW-poly(I:C) [L(I:C)], HMW-poly(I:C) [H(I:C)], or poly(dA:dT) with transfection (ng), and PMA (1 μg/mL). Cytokine levels in the supernatants were measured after 16 h of stimulation in all experiments (mean ± SD, *n* = 3). (*C*) Met was essential for TNF-α production after RNA virus infection. Parental (P) and MKO cells were infected with LCMV at the indicated multiplicity of infection (moi), and TNF-α levels in the supernatants were measured (mean ± SD, *n* = 3). (*D*) RIG-I (si-R), TLR3 (si-T), and Met (si-M) knockdown efficiency (*Left*) and TNF-α secretion (*Right*, mean ± SD, *n* = 3) in knockdown cells after stimulation. L(I:C): 100 ng. PMA: 1 μg/mL. (*E* and *F*) Attenuation of RIG-I-MAVS signalosome in MKO cells. L(I:C) 200 ng. (*E*) Western blot analysis of cell lysates. (*F*) SDD-AGE and western blot analysis of mitochondrial fraction showing MAVS oligomer. *P < 0.05. **P < 0.01. ***P < 0.001.

Met RNAi knockdown carcinoma cells and normal human epithelial cells displayed an unexpected loss in their ability to secrete TNF-α, IL-6, and IFN-β, specifically in response to short and long intracellular poly(I:C) stimulations that mimic intracellular virus dsRNA (double-stranded RNA) (8) (Fig. 1 *A* and *B*). In contrast, these cytokine production were unaltered in response to synthetic dsDNA analog poly(dA:dT) and phorbol 12-myristate 13-acetate (PMA) (Fig. 1*B*). Furthermore, Met-knockout (MKO) cells infected with lymphocytic choriomeningitis virus (LCMV) produced significantly lower levels of TNF-α and IL-6 than parental cells (Fig. 1*C*). These observations indicated that Met-deficiency resulted in a defective response to cytosolic nonself dsRNA produced by RNA virus.

Intracellular short poly(I:C) could be recognized by RIG-I and TLR3 (1, 2, 8). To elucidate the specific pathways in partnership with Met, RNAi knockdown of TLR3, RIG-I, and Met was performed (Fig. 1*D*). The knockdown of TLR3 or RIG-I each partially reduced poly(I:C)-induced TNF-α production, reflecting their concurrent involvement. Of note, TNF-α induction was completely abrogated with the double knockdown of Met/TLR3 or RIG-I/TLR3. In contrast, the combined knockdown of Met/RIG-I had no additive effects, indicating that Met and RIG-I act via the same pathway in dsRNA sensing (Fig. 1*D*). Consistent with this, poly(I:C)-induced phosphorylation of TBK1 and IRF3 (Fig. 1*E*) and MAVS aggregation (Fig. 1*F*) were significantly attenuated in MKO cells compared with their parental counterparts. Together, these observations indicated that Met is requisite for RIG-I-induced MAVS signalosome formation.

**Fig. 2.**
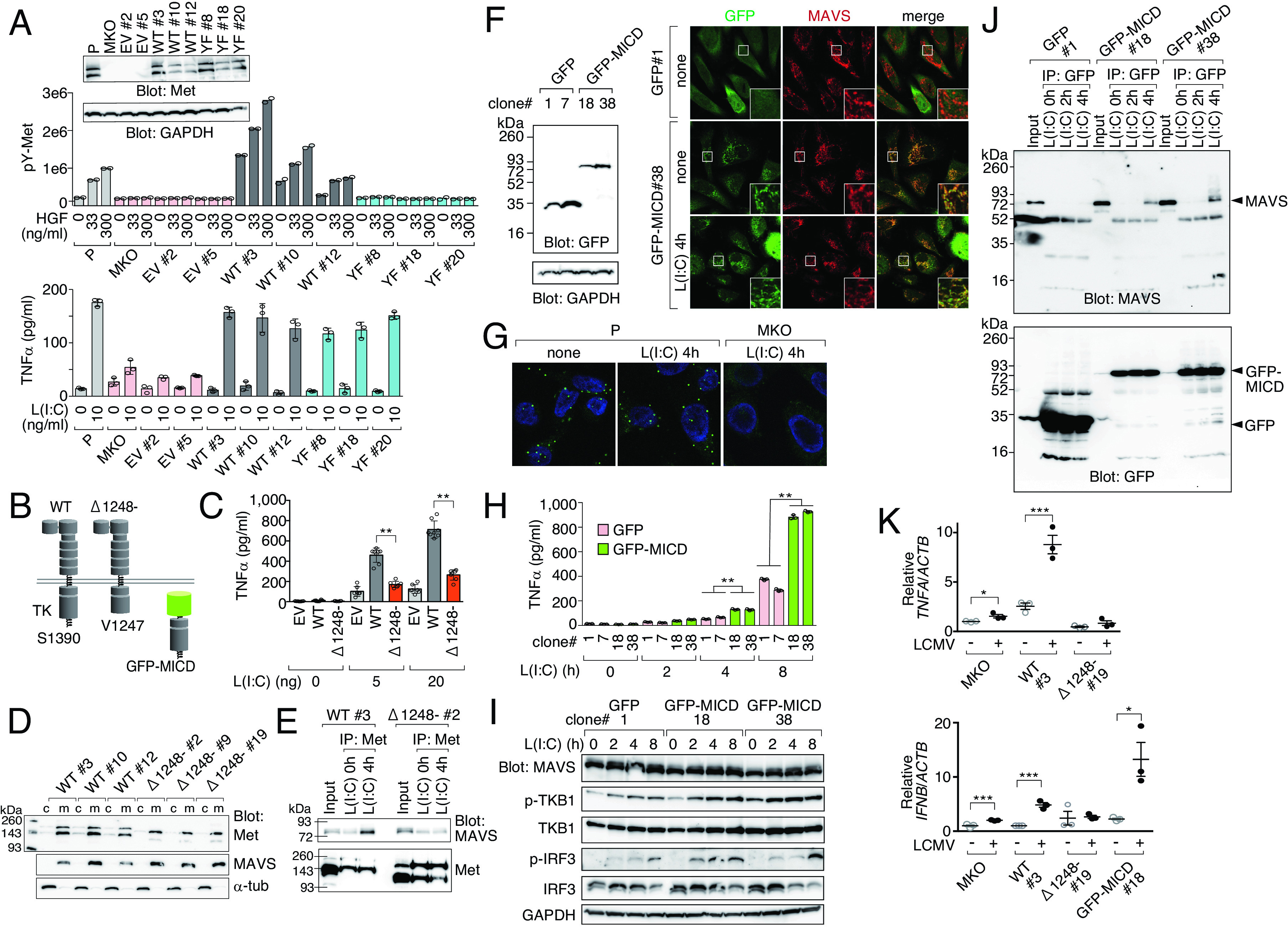
Met ICD colocalizes with MAVS on mitochondria and enhances RIG-I-MAVS signaling. (*A*) Kinase activity–independent contribution of Met to poly(I:C)-induced TNF-α production. Parental (P), MKO, and Met-restored clones [empty vector (EV), WT, or Y1234F/Y1235F Met (YF)]. Met phosphorylation at Y1234/1235 induced by HGF (*Upper*, mean, *n* = 2). L(I:C) transfection-induced TNF-α levels in the supernatants (*Lower*, mean ± SD, *n* = 3). (*B*) Schematics showing Met ICD deletion mutant (Δ1248-) and GFP-Met ICD fusion (GFP-MICD). (*C*–*E*) Carboxy-terminal Met ICD is important for interaction with MAVS. (*C*) MetΔ1248- failed to restore TNF-α secretion. MKO cells restored with EV, WT, or Δ1248-. L(I:C) transfection-induced TNF-α levels in the supernatants (mean ± SD, *n* > 6). (*D*) Met WT and MetΔ1248- localize to mitochondria. Cytoplasmic (c) and mitochondrial (m) fractions. (*E*) Met WT, but not MetΔ1248-, associates with MAVS after L(I:C) stimulation. Mitochondrial fraction was immunoprecipitated with Met and followed by western blotting for MAVS and Met. (*F*–*J*) Met ICD colocalizes with MAVS and enhances RIG-I-MAVS signaling. (*F*) GFP- or GFP-MICD-restored MKO clones (*Left*). Immunofluorescence showing GFP-MICD colocalizes with MAVS (*Right*). (*G*) Colocalization of endogenous Met and MAVS assessed by PLA. (*H* and *I*) Exogenous GFP-MICD augmented TNF-α secretion (mean ± SD, *n* = 3) (*H*) and RIG-I signaling (*I*) after 20 ng L(I:C) treatment. Cell lysates were analyzed by western blotting. (*J*) Stimulation-dependent coprecipitation of MAVS with GFP-MICD but not with GFP. Cell lysates were immunoprecipitated using anti-GFP antibody followed by western blotting for MAVS or GFP. (*K*) Real-time RT-PCR analysis of *TNFA* and *IFNB* in MKO and Met-restored clones (mean ± SD, *n* = 3, nontreated MKO was set as 1). Cells were left untreated or infected with LCMV at the 0.1 moi and total RNA was extracted 24 h after infection. *P < 0.05. **P < 0.01. ***P < 0.001.

Unexpectedly, we observed that the reconstitution of MKO cells with the kinase-defective Met Y1234/1235F mutant (YF mutant; Fig. 2 *A*, *Upper*) restored poly(I:C)-induced TNF-α production to levels comparable to that of wild-type (WT) Met and parental cells (Fig. 2 *A*, *Lower*). As the intracellular domain (ICD) of Met is known to be targeted to the mitochondria (9), we reconstituted MKO cells with a truncated Met in which the ICD was deleted from K1248 (MetΔ1248-) (Fig. 2*B*). Compared with WT Met, MetΔ1248- markedly reduced poly(I:C)-induced TNF-α production (Fig. 2*C*), suggesting the involvement of the intracellular carboxy terminus. Both WT and MetΔ1248- proteins were observed in the purified mitochondrial fraction (Fig. 2*D*) and showed partial association with MAVS (Fig. 2*E*), which was likely due to basal or nonspecific interaction. Notably, treatment with poly(I:C) significantly increased the coimmunoprecipitation of MAVS with WT Met, but not with MetΔ1248- (Fig. 2*E*). Additionally, exogenous GFP-Met ICD fusion protein (GFP-MICD, Fig. 2 *B* and *F*) colocalized with mitochondrial MAVS, independent of poly(I:C) exposure (Fig. 2*F*). The colocalization of endogenous Met and MAVS independent of poly(I:C) exposure was confirmed by proximity ligation assay (PLA) (Fig. 2*G*). Moreover, exogenous GFP-MICD markedly enhanced poly(I:C)-induced TNF-α production (Fig. 2*H*) and phosphorylation of TBK1 and IRF3 (Fig. 2*I*). MAVS was coprecipitated with GFP-MICD after poly(I:C) stimulation, but not with GFP (Fig. 2*J*). Last, we compared the effects of LCMV infection in MKO cells reconstituted with Met-WT, MetΔ1248-, or GFP-MICD. The reconstitution with Met-WT or GFP-MICD, but not MetΔ1248-, effectively restored TNF-α and IFN-β mRNA induction upon LCMV infection (Fig. 2*K*). Collectively, these data provide further evidence that Met is necessary for LCMV-induced TNF-α and IFN-β gene expression and that this is mediated via its C-terminal domain.

Taken together, we propose that mitochondrial-targeted Met functionally interacts with MAVS through the carboxy terminus to enhance RIG-I-mediated MAVS signalosome formation following exposure to cytosolic nonself dsRNA. As the Met enhancement of TNF-α production was also observed for long poly(I:C), recognized mainly by MDA5 (8), Met is implicated in a downstream mechanism common to RIG-I and MDA5. As RIG-I and MAD5 are important in immune responses against hepatitis C virus (3), influenza virus (3), and SARS-CoV-2 infection (10–12), our observation implicates a role for epithelial Met in the defense against these RNA viruses. Last, HGF-induced Met receptor activation regulates the function of dendritic cells, monocytes, and macrophages (13). It would be of interest to see if Met is functionally needed for the RIG-I/MAD5-MAVS pathway in sensing RNA viruses in these antigen-presenting cells in addition to its canonical kinase-dependent activities.

## Materials and Methods

The concentrations of cytokines were measured using ELISA MAX Standard kits for human TNF-α (BioLegend), OptEIA ELISA kit for human IL-6 (BD Pharmingen), and DuoSet ELISA for human IFN-β (R&D systems) according to the manufacturer’s protocol. Details are provided in *SI Appendix*.

## Supplementary Material

Appendix 01 (PDF)

## Data Availability

All study data are included in the article and/or *SI Appendix*.
