# Supplementary material for: Met receptor is essential for MAVS-mediated antiviral innate immunity in epithelial cells independent of its kinase activity
Source: Proc Natl Acad Sci U S A. 2023 Sep 25;120(40):e2307318120. doi: 10.1073/pnas.2307318120 (PMC10556573; doi:10.1073/pnas.2307318120)
Supplement: Supplementary file 1 — Appendix 01 (PDF) [file pnas.2307318120.sapp.pdf]

PNAS

Supporting Information for
Met Receptor is Essential for MAVS-Mediated Antiviral Innate Immunity in Epithelial Cells Independent of its Kinase Activity

Ryu Imamura*, Hiroki Sato, Dominic Chih-Cheng Voon, Takayoshi Shirasaki, Masao Honda, Makoto Kurachi, Katsuya Sakai*, and Kunio Matsumoto

*Corresponding author: Ryu Imamura, Katsuya Sakai
Email: imamura.ryu.prime@osaka-u.ac.jp, k_sakai@staff.kanazawa-u.ac.jp

This PDF file includes:

Extended Methods
SI References

Extended Methods

Materials and Cell culture. Recombinant human HGF prepared from the conditioned medium of CHO cells stably expressing human HGF was provided from Kringle Pharma Inc. LMW-poly(I:C)-[L(I:C)], HMW-poly(I:C) [H(I:C)], poly(dA:dT), Phorbol 12-Myristate 13-Acetate (PMA) (FUJIFILM Wako Pure Chemical) were purchased. The following antibodies were purchased from Cell Signaling Technology: Met (D1C2) XP rabbit mAb, Met (25H2) mouse mAb, phospho-Met (Tyr1234/1235) (D26) XP rabbit mAb, RIG-I (D14G6) rabbit mAb, Toll-like Receptor 3 (D10F10) rabbit mAb, MAVS (D5A9E) rabbit mAb, phosho-IRF3 (Ser396) (D601M) rabbit mAb, IRF3 (D9J5Q) mouse mAb, phospho-TBK1/NAK (Ser172) (D52C2) rabbit mAb, TBK1/NAK1 (E9H5S) mouse mAb, GFP (D5.1) XP rabbit mAb, GAPDH (14C10) rabbit mAb. Anti-Met (D-4) and anti-MAVS (E-3) mouse mAb were purchased from Santa Cruz Biotechnology. Anti-Met (clone 5E9C4) mouse mAb was purchased from ProMab Biotechnologies. Anti-α-tubulin (clone B-5-1-2) mouse mAb was purchased from Sigma-Aldrich. Anti-MTCO1 (1D6E1A8) mouse mAb was purchased from abcam. Anti-DDDDK-tag mouse mAb or rabbit polyclonal Ab was purchased from MBL. Biotin-conjugated goat anti-GFP mAb was purchased from Rockland Immunochemicals. Alexa Fluor 488 goat anti-rabbit IgG (HL), Streptavidin-conjugated Alexa Fluor 488, and Alexa Fluor 594 goat anti-mouse IgG (HL) were purchased from Thermo Fisher Scientific. Horseradish peroxidase (HRP)-conjugated anti-rabbit or anti-mouse goat antibody were purchased from Dako.

The HuCCT1 human bile duct carcinoma cell line was obtained from the Japanese Cancer Research Resources Bank. Human lung adenocarcinoma PC-9 cell line was kindly provided by Prof. Seiji Yano (Kanazawa University, Japan). HuCCT1 cells and PC9 cells were maintained in RPMI1640 medium (FUJIFILM Wako Pure Chemical) supplemented with 10% (v/v) fetal bovine serum (FBS) and 100 U/mL penicillin, and 100 μg/mL streptomycin (nacalai tesque). HepG2 cells were purchased from the American Type Culture Collection (Manassas, VA) and were maintained in Dulbecco's modified Eagle's medium (FUJIFILM Wako Pure Chemical) containing 10% (v/v) FBS, 100 U/mL penicillin, and 100 μg/mL streptomycin (nacalai tesque). Met KO cells based on HuCCT1 cells or PC9 cells were established as described previously (1, 2). Human renal proximal tubule epithelial cells (RPTEC) were purchased from Lonza. Normal human bronchial epithelial cells (HBEpC) were purchased from PromoCell. Cells were seeded in 96-well plates at a density of 1.5×10^4 cells per well for HuCCT1 cells or 1.0×10^4 cells per well for PC9 cells and cultured for 24 h. After stimulation (add to the medium or deliver into cells), further cultured for 16 h, supernatants were collected to measure cytokine via ELISA. In some experiments, viability of remaining cells was assessed through CellTiter 96 Aqueous One Solution Cell Proliferation Assay (Promega) according to the manufacturer's protocol.

Expression plasmids and siRNA transfection. Human Met cDNA (NP_000236.2) was inserted into the pCAGGS plasmid (3), and flag-tag sequence (GACTACAAGGACGATGACGACAAG) was connected to the 3′ end of it, designated as pCAGGS-Met-FLAG. Tyrosine residues position at 1234 and 1235 in human Met were replaced by phenylalanine, i.e., kinase defective mutant (YF mutant), expression vector: pCAGGS-MetY1234/1235F-FLAG was constructed by mutagenesis PCR using pCAGGS-hMet-FLAG as a template with mutated Y1234/1235F primers as follows, 5'- TATGATAAAGAATTCTTTAGTGTACACAAC-3' and 5'-GTTGTGTACACTAAAGAATTCTTTATCATA-3'. pCAGGS-Met-FLAG was cut by Bst XI plus Xho I following blunting modification and self-ligated to construct for pCAGGS-MetΔ1248--FLAG. Xho I fragment containing coding region for Met ICD prepared from pCAGGS-Met-FLAG was ligated into Xho I site of pEGFP-C1 (CLONTEC) to construct pEGFP-C1-Met ICD. pHygBΔDTA (a kind gift from Dr. Tetsushi Sakuma, Hiroshima University, Japan) contains the hygromycin resistance gene driven by mouse phosphoglycerate kinase 1 (PGK) promoter.

Mission siRNAs were purchased from Sigma-Aldrich as follows: Mission_SIC-001 (Mission Negative control) for control siRNA, Hs_MET_9694 (#1) and Hs_MET_9698 (#3) for Met, Mission esiRNA Human ddx58 (EHU131051) for RIG-I, Mission esiRNA Human tlr3 (EHU019541) for TLR3, Mission esiRNA Human capn1 (EHU032581) for Calpain 1, and Mission esiRNA Human capn2 (EHU025391) for Calpain 2. Cells were seeded at 1×10^4 cells per well in 96-well plates

(triplicates for each stimulation) and siRNAs (100 nM) were introduced by LF2000 according to the manufacturer's protocol. After 48 h, cells were stimulated or transfected with or without poly(I:C) using LF2000 reagent. After further 16 h incubation, supernatants were collected for ELISA and cells from triplicated wells without stimulation were mixed for whole cell extract preparation to evaluate knockdown efficacy of target molecules using Western blotting.

Western blotting and Immunoprecipitation. For Western blotting, whole cell lysates were prepared in lysis buffer (40 mM Tris, pH 7.6, 150 mM NaCl, 10 % Glycerol 1% Nonidet P-40, 1% Triton X-100, 2 mM EDTA) supplemented with protease inhibitor cocktail (nacalai tesque). Cell lysates and mitochondrial lysates were separated by SDS-PAGE and proteins were visualized by ImmunoStar LD according to the manufacturer's instructions (FUJIFILM Wako Pure Chemical). For protein-protein interactions, whole cell lysates or mitochondrial lysates were incubated with primary antibodies at 4°C overnight, followed by incubating with magnetic protein G or streptavidin beads (Dynabeads, Thermo-Fisher Scientific) at 4°C for 4 h. The beads were washed four times with lysis buffer and analyzed by Western blotting. Input: 1% mitochondrial lysate used for immunoprecipitation as an input control.

Establishment of stable cell lines. pCAGGS-hMet-FLAG (WT, YF mutant, or Met ICD deletion mutants) or pEGFP-C1-Met ICD, and hygromycin-resistant plasmid (pHygBΔDTA) were co-transfected at a 10:1 ratio into Met KO PC-9 cells in 60-mm dishes using Lipofectamine LTX with Plus transfection reagent (Thermo-Fisher Scientific) according to the manufacturer's instructions. pCAGGS or pEGFP-C1 was also transfected as an empty vector or GFP expression vector, respectively. After 24 h, cells were selected by culturing in growth medium supplemented with Hygromycin B (100 μg/mL) for 10 days. Stably expressing cells were obtained through limiting dilution and further selected by analyses through flow cytometer or Western blotting using anti-Met or anti-GFP antibody.

In situ detection of Met phosphorylation. Cells were seeded in 96-well black micro-clear plates (Greiner Bio-One) at a density of 1.5×10^4 cells per well for HuCCT1 cells or 1.0×10^4 cells per well for PC9 cells and its derivative stable clones and cultured for 24 h. The cells were stimulated with HGF in RPMI1640 medium supplemented with 10% FBS for 10 min, washed once with ice-cold phosphate-buffered saline (PBS), and fixed with 4% paraformaldehyde in PBS for 30 min at room temperature. After washing three times with PBS, the cells were blocked with 5% goat serum, 0.02% Triton X-100 in PBS for 30 min at room temperature and incubated with anti-phospho-Met (Y1234/1235) (D26) XP rabbit mAb (1:1000 diluted in PBS with 1% goat serum) at 4°C, overnight. The cells were washed three times with PBS and incubated in HRP-conjugated anti-rabbit goat antibody (1:1000 diluted in PBS with 1% goat serum) for 1 h. After washing four times with PBS, tyrosine-phosphorylated Met receptor was detected by ImmunoStar LD reagent (FUJIFILM Wako Pure Chemical) and measured using ARVO MX (Perkin Elmer).

Isolation of mitochondria from cultured cells and MAVS aggregation assay. Mitochondria from cells were isolated through the Mitochondria isolation kit (Thermo-Fisher Scientific) and mitochondrial pellet was lysed with 10 mM CHAPS in TBS (25 mM Tris, 0.15M NaCl, pH7.2) for Western blotting. For Semi-Denaturing Detergent-Agarose Gel Electrophoresis (SDD-AGE) to detect MAVS aggregation, mitochondrial pellet was suspended in 4X sample buffer (2X Tris/Acetic Acid/EDTA (TAE), 20% glycerol, 4% (w/v) SDS, and 0.01% (w/v) bromophenol blue) left standing at room temperature for 15 min and loaded to 1.7% of high-strength agarose gel (Lonza) immersed in 0.5% SDS, 1X TAE buffer and the DNA electrophoresis was performed at 100 V for 45 min in cold room, followed by western blotting using anti-MAVS or anti-Met antibody.

Immunocytochemistry and proximity ligation assay. Cells were seeded on the cover glass at 1.5×10^5 cells per well in 24-well plates and transfected with or without poly(I:C). Some samples were pre-stained with 100 nM MitoTracker Red CMXRos (Thermo Fischer Scientific) for 30 min before harvest. After 2 or 4 h stimulation, the cells were fixed with 4% paraformaldehyde/PBS for 10 min, permeabilized with 0.1% Triton X-100/PBS for 5 min, and blocked with 3% FBS/PBS for 30 min, incubated with rabbit anti-Met mAb (D1C2 CST) or biotin-conjugated goat anti-GFP mAb

(Rockland Immunochemicals), and mouse anti-MAVS mAb (E-3, Santa Cruz Biotechnology) at 37˚C for 30 min, sequentially incubated with Alexa Fluor 488-conjugated goat anti-rabbit IgG or Alexa Fluor 488-conjugated streptavidin, and Alexa Fluor 594-conjugated goat anti-mouse IgG (Invitrogen) at 37˚C for 30 min. Finally, samples were mounted in Fluorescence Mounting Medium (Dako). Images were acquired using a LSM510 META confocal microscope and its imaging system ZEN (Carl Zeiss). Proximity between endogenous Met and MAVS was assessed through proximity ligation assay using rabbit anti-Met mAb (D1C2) and mouse anti-MAVS mAb (E-3) based on DuoLink in situ GREEN kit (Sigma-Aldrich) according to the manufacturer's protocol.

Viruses. LCMV Armstrong strain (Arm) was propagated on BHK cells and titered on Vero cells as previously described (4).

RNA extraction and quantitative RT-PCR. Total RNA was isolated using a QIAzol Lysis Reagent (QIAGEN), and cDNA was synthesized with a SuperScript™ III Reverse Transcriptase (Thermo Fisher Scientific). Real-time PCR was performed using the ViiA™ 7 Real-Time PCR System (Applied Biosystems) according to the manufacturer's instructions. The primer pairs and probes for human *TNFA*, *IFNB*, and *ACTB* were obtained from the TaqMan assay reagents library (Thermo Fisher Scientific).

Statistical analysis. An unpaired Student's t-test (two-tailed) and a two-way ANOVA (multiple comparisons) were used to assess statistical significance. Prism 5 (GraphPad Software) was used to calculate statistics. Results were considered significant at a P value of < 0.05.

SI References

1. W. Miao, *et al.*, MET Activation by a Macrocyclic Peptide Agonist that Couples to Biological Responses Differently from HGF in a Context-Dependent Manner. *Int J Mol Sci* 19, (2018).
2. W. Miao, *et al.*, Impaired ligand-dependent MET activation caused by an extracellular SEMA domain missense mutation in lung cancer. *Cancer Sci* 110, 3340–3349 (2019).
3. H. Niwa, K. Yamamura, J. Miyazaki, Efficient selection for high-expression transfectants with a novel eukaryotic vector. *Gene* 108, 193–200 (1991).
4. M. Kurachi, *et al.*, The transcription factor BATF operates as an essential differentiation checkpoint in early effector CD8+ T cells. *Nat Immunol.* 15, 373–383 (2014).
